# The limitations, dangers, and benefits of simple methods for testing identifiability

**DOI:** 10.1371/journal.pcbi.1009425

**Published:** 2021-10-14

**Authors:** Mario Castro, Rob J. de Boer

**Affiliations:** 1 Grupo Interdisciplinar de Sistemas Complejos (GISC), Madrid, Spain; 2 Instituto de Investigación Tecnológica (IIT), Universidad Pontificia Comillas, Madrid, Spain; 3 Theoretical Biology and Bioinformatics, Utrecht University, Utrecht, The Netherlands; Inria, FRANCE

## Abstract

In their Commentary paper, Villaverde and Massonis (On testing structural identifiability by a simple scaling method: relying on scaling symmetries can be misleading) have commented on our paper in which we proposed a simple scaling method to test structural identifiability. Our scaling invariance method (SIM) tests for scaling symmetries only, and Villaverde and Massonis correctly show the SIM may fail to detect identifiability problems when a model has other types of symmetries. We agree with the limitations raised by these authors but, also, we emphasize that the method is still valuable for its applicability to a wide variety of models, its simplicity, and even as a tool to introduce the problem of identifiability to investigators with little training in mathematics.

In their Commentary paper, Villaverde and Massonis (*On testing structural identifiability by a simple scaling method*: *relying on scaling symmetries can be misleading* [1]) have commented on our paper in which we proposed a simple scaling method to test structural identifiability [2]. Our scaling invariance method (SIM) tests for scaling symmetries only, and Villaverde and Massonis correctly show the SIM may fail to detect identifiability problems when a model has other types of symmetries (we indeed indicated but not investigated the importance of generalizing the method to other symmetries). Thus, we agree that our simple method provides a necessary but not sufficient condition for identifiability, and we appreciate their careful analysis and constructive criticism.

We nevertheless think that the simple method remains useful because it is so simple. Even for investigators with little training in mathematics, the method provides a necessary condition for structural identifiability that can be derived in a few minutes with pen and paper. Similarly, we have found its pedagogic strength by teaching the method to our own graduate students and colleagues. More advanced methods (such as STRIKE-GOLDD [3,4], COMBOS [5], or SIAN [6]) are typically intimidating for researchers with a background in Biology or Bioinformatics. This simple method can help those practitioners to familiarize themselves with the identifiability problem and better understand their models.

Finally, it is worth noting that if scaling invariance is the only symmetry (as it was in all the cases we analyzed), our SIM remains valuable (albeit uncontrolled), and surprisingly effective for a wide variety of problems (as the extensive list collected in the Supplementary Material our paper [2]). We guess that the SIM especially fails when applied to linear models (as more potential *rotations* of the variables leave the system invariant), and in non-linear scenarios where some parameters are identical. For instance, the FitzHugh-Nagumo model raised by Villaverde and Massonis,

$$\begin{array}{ll} {\dot{x}}_{1}\left(t\right) & =c\left(x_{1}\left(t\right)-\frac{x_{1}^{3}\left(t\right)}{3}-x_{2}\left(t\right)+d\right),\\ {\dot{x}}_{2}\left(t\right) & =\frac{1}{c}\left(x_{1}\left(t\right)+a-b\cdot x_{2}\left(t\right)\right),\\ \;y\left(t\right) & =x_{1}\left(t\right), \end{array}$$

could have been written as

$$\begin{array}{ll} {\dot{x}}_{1}\left(t\right) & =\lambda_{1}x_{1}\left(t\right)-\lambda_{2}\frac{x_{1}^{3}\left(t\right)}{3}-\lambda_{3}x_{2}\left(t\right)+d,\\ {\dot{x}}_{2}\left(t\right) & =\lambda_{4}x_{1}\left(t\right)+a-b\cdot x_{2}\left(t\right),\\ \;y\left(t\right) & =x_{1}\left(t\right) \end{array}$$

where *λ*_1_ = *λ*_2_ = *λ*_3_ = 1/*λ*_4_ = *c*. One of the reasons why our method fails, in this case, might be these additional symmetries introduced in this more elaborate notation of the model.

Hence, it is worth understanding generic conditions under which the SIM method is expected to be fragile, possibly using STRIKE-GOLDD to test large families of nonlinear models.

As a final remark, we appreciate that Villaverde and Massonis have shared their source code, so researchers might have a *gold standard* to test identifiability.
